# Cellulose-Based Metallogels—Part 2: Physico-Chemical Properties and Biological Stability

**DOI:** 10.3390/gels9080633

**Published:** 2023-08-07

**Authors:** Aleksandra Mikhailidi, Irina Volf, Dan Belosinschi, Bogdan-Marian Tofanica, Elena Ungureanu

**Affiliations:** 1 Higher School of Printing and Media Technologies, St. Petersburg State University of Industrial Technologies and Design, 18 Bolshaya Morskaya Street, 191186 St. Petersburg, Russia; amikhailidi@gmail.com; 2 “Gheorghe Asachi” Technical University of Iasi, 73 Prof. Dr. Docent D. Mangeron Boulevard, 700050 Iasi, Romania; 3 Département de Chimie-Biologie/Biologie Medicale, Université du Québec à Trois-Rivières, Trois-Rivieres, QC G8Z 4M3, Canada; dbelosinschi@gmail.com; 4 IF2000 Academic Foundation, 73 Prof. Dr. Docent D. Mangeron Boulevard, 700050 Iasi, Romania; 5 “Ion Ionescu de la Brad” University of Life Sciences Iasi, 3 Mihail Sadoveanu Alley, 700490 Iasi, Romania; eungureanu@uaiasi.ro

**Keywords:** metallogels, hydrogels, cellulose, physico-chemical properties, biological stability

## Abstract

Metallogels represent a class of composite materials in which a metal can be a part of the gel network as a coordinated ion, act as a cross-linker, or be incorporated as metal nanoparticles in the gel matrix. Cellulose is a natural polymer that has a set of beneficial ecological, economic, and other properties that make it sustainable: wide availability, renewability of raw materials, low-cost, biocompatibility, and biodegradability. That is why metallogels based on cellulose hydrogels and additionally enriched with new properties delivered by metals offer exciting opportunities for advanced biomaterials. Cellulosic metallogels can be either transparent or opaque, which is determined by the nature of the raw materials for the hydrogel and the metal content in the metallogel. They also exhibit a variety of colors depending on the type of metal or its compounds. Due to the introduction of metals, the mechanical strength, thermal stability, and swelling ability of cellulosic materials are improved; however, in certain conditions, metal nanoparticles can deteriorate these characteristics. The embedding of metal into the hydrogel generally does not alter the supramolecular structure of the cellulose matrix, but the crystallinity index changes after decoration with metal particles. Metallogels containing silver (0), gold (0), and Zn(II) reveal antimicrobial and antiviral properties; in some cases, promotion of cell activity and proliferation are reported. The pore system of cellulose-based metallogels allows for a prolonged biocidal effect. Thus, the incorporation of metals into cellulose-based gels introduces unique properties and functionalities of this material.

## 1. Introduction

Gels are described as spatially structured semi-solid 3D polymer networks that are expanded throughout the whole volume by a gas or a fluid. Aerogels are formed after the fluid is removed and substituted by air [1], while hydrogels are filled with liquid as the medium [2,3,4,5,6]. Thus, a gel can be seen as a large amount of solvent immobilized by a small amount of crosslinked solid [3]. If a metal is a part of the gel network as a coordinated ion, as a cross-linker, or as metal nanoparticles adhered to the gel matrix, it is called a metallogel [7,8].

Metallogels can be considered colloidal compounds that do not dissolve but can diffuse through membranes and can be included in the category of soft composite materials. These materials borrow from the properties of the initial polymer, as well as of the metal that can complex with the polymer and form stable chelated compounds with improved properties, such as adsorption capacity [9,10], optical or magnetic response activity, conductivity, catalytic activity, biocidal properties, etc. [3,9,11,12,13]. In this regard, metallogels based on natural polymers, for instance, lignin [14,15], chitosan [16,17], or cellulose [18,19], which have such industrially favorable properties, such as wide availability, low cost, renewability, biocompatibility, and biodegradability, are of particular interest [20].

By harnessing the unique properties of cellulose and incorporating metals into the gel matrix, cellulose-based metallogels offer exciting opportunities for advanced biomaterials and new functional applications.

Cellulose can be separated from different plant-based biomass sources and converted through chemical, thermochemical, or biochemical processes [21,22], including the following:

- Wood pulp is a common and abundant source of cellulose. It can be obtained from different types of trees and processed to extract cellulose fibers, which can then be used to produce cellulose [23].
- Plant-based fibers come from various plants, such as cotton, bamboo, jute, hemp, and flax, and contain up to 99% of cellulose in their cell walls [24]. Fibers from these plants can be processed to extract cellulose.
- Agricultural waste contains large quantities of annually renewed cellulose that can be isolated and extracted from rice husks, sugarcane bagasse, wheat straw [25], corn stalks [26], rapeseed stalks [27], and others. These waste materials can be processed for various applications, contributing to the sustainable utilization of agricultural by-products.
- Algae biomass is easily obtained, requires a reduced number of operations for preparation [28,29], and certain species contain cellulose and can be a potential source for cellulose extraction [30,31,32].
- Bacteria and microorganisms: cellulose-producing bacteria and microorganisms, for instance, *Acetobacter xylinum*, can be used to produce cellulose through fermentation processes [33].
- Waste paper is another significant source of cellulose [34]. However, compared to other sources, waste paper may require additional extraction steps due to its heterogeneous composition and impurities resulting from the papermaking process. The recycling of waste paper involves deinking and pulping processes to remove inks, coatings, and other contaminants before cellulose fibers can be obtained.

Cellulose is a natural polysaccharide that is in high demand in many industries; it is presented almost in every sphere of human life, starting from the construction sector [35], paper and packaging [36], textile industries [37], agriculture [38] and food production [39], chemical and oil industry [40], and wastewater treatment [41]. Biomedicine [42], cosmetics and healthcare [43,44], and electronic devices [45] need cellulosic materials as well.

New uses of cellulose, the old raw material, are the production of hydrogels [46,47]. The majority of research projects aim to produce hydrogels not from a natural (plant-derived) cellulose but from its derivatives, such as ethers and esters (carboxymethyl cellulose [48], hydroxyethyl cellulose [49], methylcellulose [50]), or after crosslinking with poly(acrylic acid) [51], poly(vinyl alcohol) [52], poly(ethylene glycol) [53] and poly(N-vinylcaprolactam) [54].

Enhanced properties of hydrogels, such as improved stimuli-responsiveness (pH, temperature, redox, chemicals, UV illumination), improved mechanical characteristics, and self-healing ability, are achieved through derivatization, oxidation, crosslinking, and/or the incorporation of nanocellulose [3,48,49,55,56]. However, derivatization is usually a complex time-, energy, and resource-consuming procedure which requires additional steps; additionally, it is not always suitable for a productional scale.

The choice of cellulose source for hydrogel production depends on several factors, such as availability, cost, sustainability, and desired properties of the hydrogel. Each cellulose source may have its own unique characteristics, which can impact the properties and applications of the resulting hydrogel materials.

Non-derivatized or unmodified cellulose or bacterial cellulose (BC) can also be transformed into hydrogels and then converted to metallogels. However, there are much fewer publications about unmodified cellulose hydrogels and metallogels on their basis. The methods of fabrication of the cellulose-based metallogels and the raw cellulosic materials for that purpose were reviewed by the authors in Part 1 of the series of publications [57]. There can be several approaches for obtaining a natural cellulose metallogel.

**Figure 1:**
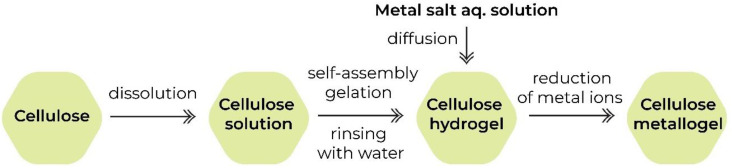
Schematic route of cellulose metallogel formation via diffusion-reduction method.

The first one is a two-step process in which, firstly, a hydrogel is obtained, and after that, metal nanoparticles are synthesized in it via the diffusion-reduction method (Figure 1). This method includes diffusion of the metal ions from the aqueous solutions of their salts into the matrix of the ready-made hydrogel and the reduction of the ions to zero-valent metal or to the metal oxides with the help of different reducers. The system of pores in cellulose limits the agglomeration of metal nanoparticles and promotes their fixation both on the surface and in the inner layers of the cellulose material [34,57,58].

Another technique for the preparation of cellulose metallogels is to add metal ions into the polymer solution before the gelation occurs. In this approach, metal ions (e.g., Ag^+^) coordinate with negatively charged surface carboxylate groups of cellulose and induce gelation, during which they are reduced to Ag(0) nanoparticles by the functional groups of cellulose without a reducing agent [59,60,61]. Moreover, the ready-made dried hydrogels can be introduced into the suspension of nanoparticles to obtain a composite metallogel by impregnation [62].

**Figure 2:**
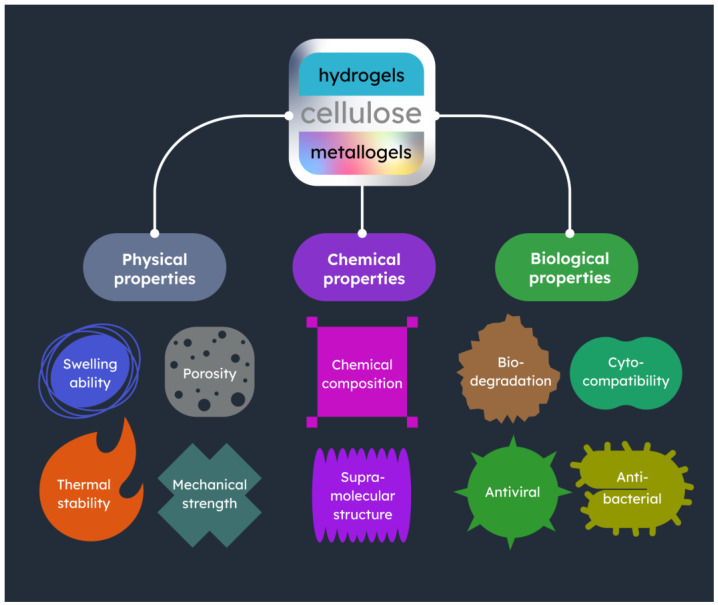
Key characteristics of the cellulose-based metallogels presented in this review.

On this basis, this review provides an overview of the properties exhibited by composite materials, which are composed of cellulose and a metal component (Figure 2). The unique characteristics of these compounds arise from the combination of the polymer and the metal component; hence, it is important to understand the properties of cellulose hydrogels from different sources to better estimate the enhancements brought by the incorporation of metals, as discussed in the relevant literature.

By doing so, we can gain insights into the synergistic effects and unique attributes that emerge from the combination of cellulose and metal in the metallogel composite materials. These investigations will shed light on the enhanced properties and potential applications of metallogels, highlighting the advancements in this field and paving the way for further research and development.

## 2. Physical Properties

### 2.1. Appearance

**Figure 3:**
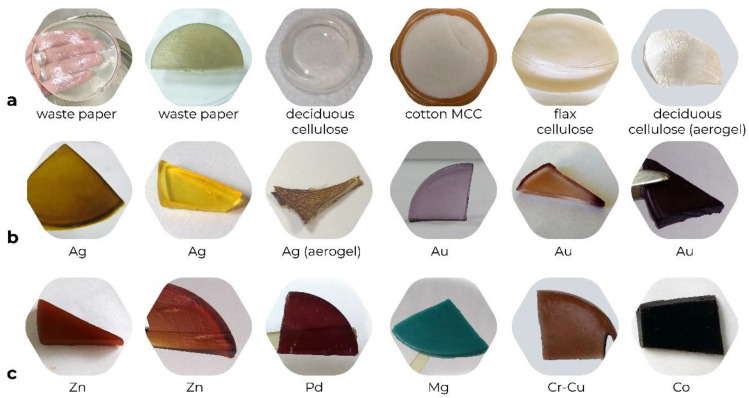
The digital photographs of the hydrogels prepared from cellulose of various origins (**row a**) and cellulose metallogels with Ag(0), Au(0) (**row b**), and with organic-metal complexes of transition metals: Zn, Pd, Mg, Cr-Cu, Co (all II valent) (**row c**).

Commonly, cellulose hydrogels have fixed-flexible sheet forms. They are also water-saturated (moist), soft, smooth, transparent, and colorless substances [58,63]; however, the color and transparency may be different depending on the initial cellulosic material (Figure 3a). For example, hydrogels obtained from cotton microcrystalline cellulose (MCC) are white-colored, hydrogels from flax cellulose perform sandy-beige color, and both are completely opaque, while cellulose hydrogels fabricated from waste paper are colorless or yellow, grey, light-brown and were either transparent or semi-clouded [34,64].

Hydrogels formed from bacterial and deciduous cellulose are transparent and colorless, although the latter may acquire a yellowish tone or become white and semi-opaque depending on the conditions of the dissolution [58,62,65]. Along with the other physical properties of hydrogels, transparency is desirable for a biomedical application, for example, for a wound dressing, since it allows one to control the wound condition without removing the dressing. As a result, the wound will not be traumatized, and the risk of infection will decrease [66].

Cellulose-based metallogels borrow the color of the introduced metal (Figure 3b,c); for instance, Ag(0) containing hydrogels have the color from light yellow to dark brown [58,59,61,62,65,67], Au(0) composite gels may have purple, pink, brick or red coloration (Figure 3b) [67,68,69,70], Pt(0)-containing metallogels are colored in black [67], while calcium based composites are white [17], reflecting all the spectrum of the visible light. The metallogels with organic-metal complexes of transition metals reveal brown or purple-brown color for Zn(II), Pd(II), and Cr(II)-Cu(II); Co(II) containing composites are almost black, and the ones with Mg(II) are colored in blue (Figure 3c) [71].

In the series of publications [58,68,72], the composites of cellulose hydrogels with Ag(0) and Au(0) nanoparticles fabricated via the diffusion-reduction method (Figure 1) were studied. It was reported that the intensity of their coloration was in accordance with the aspect ratio between cellulose and metal ions. Generally, the higher the concentration of metal ions in the gel, the more intense the color of the obtained composite becomes. Moreover, the aspect ratio between metal ions and the chemical reducer plays a role in the reduction process, contributing to the resulting metal content and the color of the hydrogels. For instance, the aspect ratio between citrate ion and Ag^+^ was reported to be (1–10):1 mol, respectively [72].

On the contrary, when the nanoparticles of Au(0) were synthesized in the hydrogel without a chemical reducer due to the reducing ability of the end aldehyde groups of cellulose chains, it was reported that the more Au-ions were introduced in the solution, the slighter the coloration of the metallogel became after reaching the saturation threshold [68]. The authors explained this fact by the limited number of the end-reducing groups in cellulose capable of interacting with the growing number of Au ions. As a result, not all of Au^+^ were involved in the redox process.

The swelled cellulose hydro- and metallogels are stable while storing in water or humid environment; unlike synthetic hydrogels, they spontaneously lose the moisture when stored in air [64]. Therefore, the swelled hydrogels are sometimes undergone by freeze-drying for some applications. Although for the majority of the hydrogels the reswelling ability was reported [73,74,75], the other cellulose hydrogels crucially collapse while drying, and form slightly accessible structures [64,76] due to the collapse of the H-bonds formed. This is a challenge for the future improvements.

Due to the ability to exhibit strong light absorption, efficient energy transfer, and excellent charge transport makes cellulose-metal complexes play a crucial role in various fields, particularly in the realm of optoelectronics technologies, making them highly valuable for a range of applications such as photovoltaic devices, light-emitting diodes (LEDs), displays, but also sensitive and selective biosensors for different analytes, including biomarkers, pathogens, and pollutants [77,78].

### 2.2. Swelling Ability

The cellulose hydrogels are super-swollen systems and retain large amounts of water, dozens of times their own dry weight [42,79,80]. The swelling of hydrogels is influenced by the available space within the network structure capable of accommodating, absorbing, and maintaining water molecules due to their hydrophilic nature [81]. The swelling behavior of hydrogels in water or biological fluids is a crucial parameter determining their properties and applications [42]; for example, the hydro- and metallogels can serve as wound or burn injuries dressings, effectively hydrating dry wound beds and promoting the softening and loosening of slough and necrotic wound debris. The hydrogels exchange moisture with dry wounds, thereby facilitating autolytic debridement and maintaining a moist wound environment that is thermally insulated. They have been shown to promote granulation and epithelialization and reduce the temperature of a wound bed by up to 5 °C [63]. Metallogels preserve these favorable properties and can be utilized as wound dressings too. Moreover, when specific metals (Ag, Au, Zn) are used, they also confer antibacterial activity to these materials [82,83].

Since the majority of the methods for obtaining cellulose-based metallogels involve ready-made hydrogels as matrices for embedding metal nanoparticles, we suppose that analysis of the cellulose hydrogels’ swelling ability can improve the understanding of their capabilities. Table 1 presents the properties of non-derivatized cellulose hydrogels obtained by dissolving in organic solvents or in the alkali/urea/water systems. A detailed analysis of the cellulose-based hydrogel preparation using these solvents is provided in the first part of the review [57]. Swelling in water is usually calculated according to Formula (1) and is given in the articles as a percentage or in grams of water per gram of dry hydrogel (g/g). For ease of comparison, we converted all values to g/g, dividing the percentage results by 100.

*EWC* = (*m_h_* − *m_d_*)·*m_d_*^−1^, (1)

where *EWC* is for the equilibrium water content, *m_h_* is the mass of the swollen hydrogel at the equilibrium, and *m_d_* is the mass of the dried specimen to a constant weight in the vacuum [64].

Even though the formula is quite simple, there are several discrepancies in its use. Firstly, drying of hydrogels in the vacuum is not mandatory; in some papers, the method of drying is not specified (e.g., in [84]); as a result, they might be dried in air or by oven heating, which obviously affects the result. Secondly, in some studies, the weight of the initial swollen hydrogel was measured, then it was dried to calculate the swelling ability [64,84], while in the other cases, the hydrogel was dried, then it reswelled in water until equilibrium, and after that, it was weighted. Initial swelling after formation and reswelling after drying may not be the same. Finally, in several studies, the authors changed the Formula (1) as the following:

*EWC* = (*W_h_* − *W_d_*)·*W_d_*^−1^, (2)

where *EWC* is for the equilibrium water content, *W_h_* is the weight of the hydrated sample, and *W_d_* is the dry weight of the sample [74].

The studies [74,85] attribute the mass of water not to a dry hydrogel but to a swollen one (Formula (2)), so their results cannot be compared to those obtained by Formula (1). Thus, to compare the equilibrium water content (swelling ability, swelling properties, water content, swelling ratio, etc.) of the hydrogels in different studies, the protocol of this measurement should be carefully checked.

Table 1 does not cover all the results obtained in recent years, but it is provided for the purpose of understanding the potential for swelling, as it presents different solvents and different starting cellulosic materials (cellulose, lignocellulose, wastes). It should be noted that Table 1 lists not only hydrogels but also hydrogel films [74,86], for which the swelling properties are lower.

The study by Tovar-Carrillo and co-authors allows one to compare hydrogels obtained in different solvent systems within the same raw material. Thus, the result revealed that of the three solvents, namely NaOH aqueous, NaOH/urea, and N,N-dimethylacetamide (DMAc)/LiCl, the latter one provided the maximum swelling ability (0.31 g/g) of the hydrogel, while for the alkali-based methods, the swelling abilities of the hydrogels were lower (0.11 and 0.14 g/g, respectively) [74]. These data are methodologically comparable with the swelling ability of the hydrogels obtained from okara fibers (0.88–0.94 g/g depending on the epichlorohydrin (ECH) content which was a cross-linker in the synthesis) using LiOH/urea/water dissolving system [85]. However, in this study, the authors added ECH as a chemical cross-linker, while in [74], all the hydrogel films were physical hydrogels.

Among the swelling abilities calculated by Formula (1), the highest result (66.75 g/g) was reported for a composite hydrogel produced from waste paper and acrylamide in NaOH/urea aqueous system with the cross-linker ECH [87]. An identical dissolving system was applied for waste paper, but the swelling abilities of the hydrogels without acrylamide in the composition of the hydrogel obviously fell drastically to 13.4–27.7 g/g compared with the previous example [88].

Acrylates are well-known agents for the improvement of the swelling properties of cellulose hydrogels in order to produce super-adsorbents [89]. For instance, the swelling ability of the hydrogel prepared from maleylated cotton stalk cellulose and acrylic acid was reported to be 1125 g/g [90]. Another superabsorbent hydrogel obtained from flax yarn waste and poly(acrylic acid-co-acrylamide) revealed a swelling ability of 875 g/g [91]. The conclusion is that acrylates improve the swelling properties of cellulose-based hydrogels significantly.

The swelling behavior of hydrogels is governed by several factors, including the polymer composition, degree of polymerization, degree of crosslinking, cellulose concentration in the initial solution, cellulose origin (Table 1), and external stimuli. The hydrogels from the solutions of DMAc/LiCl had comparatively high swelling abilities (11.53–28 g/g) depending on the type of initial material. The hydrogels from hardwood lignocellulose had the highest swelling ability, while the sugarcane bagasse hydrogels had the lowest [64,84]. However, the swelling abilities of the hydrogels obtained from agricultural wastes in dimethyl sulfoxide (DMSO)/LiCl, namely thanaka tree heartwood, sugarcane bagasse, and rice straw celluloses, were relatively similar (1.66–1.89 g/g) [86]. The lower values can be explained by the shape of the hydrogels and hydrogel films prepared in the study, in which swelling abilities were much lower for hydrogels resulting from the (DMSO)/LiCl dissolving system than for those resulting from the DMAc/LiCl.

Several studies [73,88,92] compare the swelling abilities of the hydrogels with different concentrations of lignocellulosic solutions. In general, the higher the concentration of cellulose, the bigger the swelling ability of the hydrogel; however, after a certain value of 5% [73,88], it decreases. It is connected to the porous structure of the hydrogel, which allows water to enter the hydrogel, leading to its swelling. As fewer pores are available in the hydrogel samples of a lower percentage of cellulose, such hydrogels demonstrated lower swelling properties. When the cellulose content increases, its chains are easily self-entangled with intra and intermolecular hydrogen bonds, and less space is available for pore swelling [73].

Moreover, high concentrations of cellulose cause the formation of a higher number of physical entanglements, which act as crosslinking points, and eventually reduce the hydrogel swelling ability [93]. Not only cellulose but lignin content as well influenced the swelling ability of hydrogels. Thus, the water content of the hydrogel films increased from 11.53 to 15.25 g/g with a decrease in lignin content from 1.62 to 0.68% (Table 1) [84]. Interestingly, the opposite result was reported by Liu and co-workers [94], when with the decrease of lignin from 23.24% to 14.24%, the swelling ability also fell from 22.87 to 13.96 g/g with the extremum at 7.71 g/g corresponding to 18.91% of lignin. The non-linear trend (Table 1) was explained by changes in the three-dimensional structure of the polymer caused by delignification, pore sizes, and distribution. Liu et al. revealed the dependence of the degree of hydrogel swelling on temperature. Once the temperature rises, the degree of swelling decreases, which is associated with the compression of the three-dimensional structure from heating.

We assume that the swelling ability of cellulose-based metallogels depends on the swelling ability of cellulose hydrogels. Song and collaborators studied the swelling ability of composite polyvinyl alcohol/bacterial cellulose (PVA/BC) hydrogels resulting from the dissolution in a NaOH/urea/H_2_O system, with the same one with Ag particles. It was demonstrated that metal particles increased the swelling ability of the hydrogel from 13 to 16 g/g due to pore enlargement [75]. The opposite result was reported when hybrid composite hydrogels were synthesized by immobilization of a 1,10-phenanthrocyanines Zn(II) complex into the cellulose hydrogels [71]. The swelling ability of the composite hydrogels with Zn(II) organic complexes decreased compared to the hydrogels, namely from 28.00 g/g to 14.80 g/g and from 25.00 g/g to 12.40 g/g for the hydrogels obtained from deciduous and flax cellulose, respectively. The authors explained this distinction using the differences in the chemical and supermolecular structure of the original and composite samples. Another research group reported that during the formation of the bacterial cellulose metallogel with Ni(0) and Cu(0), the swelling-induced adsorption process could control the metal size and dispersion simultaneously [95]. Thus, the swelling ability of the hydrogel determines not only the applications of the product but may also be an important condition of metallogel production.

External stimuli factors, such as pH, temperature, light, electrical fields, and magnetic fields, or the presence of certain ions, can also impact hydrogel swelling. Some hydrogels exhibit stimuli-responsive behavior, where changes in external conditions trigger volume changes. For example, temperature-responsive hydrogels may undergo swelling or deswelling upon exposure to temperature variations, while pH-responsive hydrogels can swell or shrink depending on the acidity or alkalinity of the surrounding environment [96].

To conclude this part, the swelling ability of the hydrogels obtained from the solutions of organic and inorganic solvents vary from 1.66 to 66.75 g/g, depending on the following factors: additives (e.g., acrylamide or metal particles), the dissolving system, the cross-linkers (chemical or physical hydrogels), the concentration of lignin and cellulose in the solution, pore size and distribution, temperature and the origin of the initial material.

### 2.3. Porosity

Cellulose has a 3D-network structure [97]. Especially after freeze-drying, the cellulose hydrogels reveal a developed system of pores [64], which is why they are used as porous skeletons to imitate the roles of extracellular matrices and to engineer different tissue types [98]. The swelling ability of the hydrogels depends on the type and size of the pores. The rate of swelling can be controlled by designing a system of interconnected macropores to obtain a faster reaction and vice versa [99]. The hydrogel with large pores could not retain a large amount of water, and water absorption was low [100]. The size of the pores determines the capability to act as a drug carriage in drug-delivery systems [99], as well as the absorption ability of the hydrogels when they are used as adsorbents [101].

The proportion of hydrogel volume occupied by the pores (porosity) is calculated according to the following equation:

*P_t_* = 1 − *m*_0_·*P*^−1^·*L*^−1^·*ρ^−^*^1^, (3)

where *m*_0_ is the weight of the dried hydrogel, g, *P* is a square of the surface of the hydrogel, cm^2^, *L* is the thickness of the hydrogel, cm, and *ρ* is the density of cellulose (*ρ* = 1.561 g∙cm^−3^) [71].

The porosity of plant-derived cellulose hydrogels was in the range from 86.8% (cotton) to 98.9% (flax), according to [64] (Table 1). The hydrogels obtained from waste paper demonstrated the same value of 98.1% [87]. The average size of the pores increased with the rise of the lignin content in the native lignocelluloses reaching a maximum value of 20.281 nm (Table 1) [94].

Introduction of a metal phase into the hydrogel decreases the average pore size; for instance, after the formation of the metallogel with Zn(II) organic complex, the porosity of the composite hydrogels fell to 95.8% and 94.8% compared with those of the flax and deciduous hydrogels listed in Table 1, row 3 [71]. It was also mentioned in the same study that the surface of the hydrogels was loose, and the inner structure of the hydrogels was composed of randomly distributed pores that formed a cellular honeycomb-like structure. As a result, the metallogels retained great amounts of water [71].

### 2.4. Mechanical Properties

Mechanical characteristics, namely mechanical strength and flexibility, determine the consumer properties of the hydrogels [102]. For instance, if a hydrogel is applied as a wound dressing or as a wearable sensor, it should conform to the movements of the human body. The tensile strength and elongation of the selected cellulose hydrogels are presented in Table 1. The hydrogel films produced from the solution of bamboo fibers in DMAc/LiCl revealed a tensile strength as high as 66 N/mm^2^ with a 33% elongation, while the hydrogels that resulted from NaOH solutions had lower characteristics of 21–27 N/mm^2^ and elongation of 8–13% [74]. The tensile strength of the hydrogel films containing trace amounts of lignin decreased from 0.77 to 0.43 N/mm^2^ depending on the duration of NaOH treatment and bleaching, and the relative elongation decreased from 43.8 to 26.5% with a decrease in the trace amount of lignin. The longer exposure time in the NaOH solution reduced the elasticity of the gel. The higher lignin content strengthened the hydrogel films, whereby they showed higher tensile strength and elongation [84]. The relatively low elongation of 9–35.71% demonstrated that the hydrogel films obtained from the agricultural wastes, such as thanaka tree heartwood cellulose, sugarcane bagasse cellulose, and rice straw cellulose, regenerated from DMSO/LiCl solutions [86]. The value for the hydrogel from sugarcane bagasse is two times lower than the same one obtained from the solution of DMAc/LiCl [84]. The lowest stress-at-break was reported for PVA/BC hydrogels manufactured from a NaOH/urea/H_2_O solvent system. However, the same hydrogels had the highest elongation, up to 160%.

Generally, the loading of the hydrogels with metal nanoparticles enhances their mechanical strength. Ovalle-Serrano et al. reported the dependence of stiffness of Ag(0) metallogels fabricated from cellulose nanofibers on the molar ratio between reducing functional groups COO^−^ of cellulose to AgNO_3_. High COONa:AgNO_3_ molar ratios (1:3) produced stiffer hydrogels at the expense of large cubic Ag nanoparticles clusters formation (1 µm), while lower COONa:AgNO_3_ molar ratios (1:1) resulted in softer hydrogels with spherical Ag(0) nanoparticles exhibiting diameters between 15 and 80 nm [61].

Both tensile strength and elongation of the PVA/BC-Ag metallogel improved compared to the PVA/BC hydrogel due to the crosslinking between PVA and BC by silver nanoparticles [75]. This fact is in accordance with article [83], where the authors claimed the improvement of the mechanical properties of the hydrogels due to the addition of Ag(0) nanoparticles. However, the values of stress-at-break and maximum elongation-at-break first increased and then decreased with the rise in the amount of Ag(0) nanoparticles. The authors explained the deterioration of the mechanical properties by agglomeration caused by a high concentration of silver nanoparticles, which affected the formation of a hydrogel network [75]. The same result was reported for a hydrogel obtained from a cellulose derivative when the mechanical properties of carboxymethyl cellulose hydrogels were reinforced via the incorporation of Fe^3+^. The increase of Fe^3+^ content at low iron ions concentrations formed more tridentate coordinates with the carboxyl groups of the hydrogels [103]. However, the iron ions at high concentrations transformed tridentate into monodentate or bidentate, which deteriorated the toughness of the hydrogels.

Thus, cellulose hydrogel films revealed the following mechanical properties: elongation up to 45.2% and tensile strength up to 66 N/mm^2^. The rise in strength was facilitated by an increase in the content of lignin and the addition of chemical cross-linkers or metal nanoparticles to a certain concentration. Metallogels have better mechanical characteristics compared to cellulose hydrogels; however, there is a certain limit on the metal content, after which the deterioration of the tensile strength and elongation occurs.

### 2.5. Thermal Stability

The thermal properties of materials refer to their behavior and characteristics in response to changes in temperature. These properties play a crucial role in various applications, ranging from engineering and construction to electronics and biomaterials science. Among thermal conductivity, thermal insulation, thermal expansion, thermal shock resistance, melting point, and specific heat capacity, thermal stability is essential for selecting appropriate materials for specific applications, predicting material behavior under different temperature conditions, and ensuring overall safety and performance. Thermal stability refers to a material’s ability to resist chemical or physical changes when exposed to high temperatures, decisive in applications involving elevated temperatures or thermal processing, as it ensures the material’s integrity and functionality. The thermal properties of materials can be measured using thermogravimetric analysis (TGA).

The thermal behavior of cellulose is the following. Firstly, the slight weight loss at 150 °C is attributed to the vaporization of water trapped in the cellulose materials, then the major weight loss between 250 °C and 400 °C is for the breaking off of glycosidic bonds within the cellulose chain, and finally, the residue at about 600 °C is due to the formation of a high amount of char [104]. The thermostability of the hydrogels is getting better with the cellulose content increasing with the highest decomposition temperature values from 342 °C to 352 °C (Table 1) [92]. It is related to the increase in cellulosic OH groups that contribute to hydrogen bonding. The number of hydrogen bonds between the molecules rises, and this enhances the intermolecular forces so that the thermostability of the cellulose hydrogels is improved. The listed results are obtained for the hydrogels regenerated from NMMO solutions [92]. Lower thermal stability demonstrated the hydrogels obtained via dissolution in a NaOH/urea aqueous system. The heat resistance was up to 250 °C; after that, the weight rapidly decreased due to the dehydration and depolymerization of the hydrogel. The derivative thermogravimetric (DTG) curve revealed a broad peak between 300 °C and 400 °C [73]. The additive of acrylamide increased the thermostability of the cellulose hydrogels obtained from waste paper in the same solvent system up to 407.72 °C [87]. The thermal stability of cellulose films regenerated from the solution of inorganic molten salt hydrates was from 231 °C to 307 °C, depending on the salt concentration [105].

TGA analyses demonstrated that Ag(0), as well as Zn(0) nanoparticles, increased the thermal stability of the metallogel compared to the unmodified hydrogel [61,106]. In some cases, it was reported that thermal stability was firmly preserved for the obtained composites Ag(0)-cellulose [107]. There were characteristic differences in the influences of metal species embedded into cellulose aerogels on their thermal destruction in air and in nitrogen [67]. The presence of silver nanoparticles (5 wt.%) caused an increase in the remaining char at 600 °C according to the level of silver deposition. In nitrogen, the Au(0)-cellulose specimen gave a significantly higher char yield at 600 °C than Pt(0)- and Ag(0)-cellulose. The authors explained this fact by a specific interference of gold on cellulose decomposition, which might be attributed to the catalytic effect of gold altering some stage of cellulose decomposition. By contrast, under air, the acceleration of cellulose combustion by metal particles occurred. With Ag(0) and Au(0), the secondary stage of cellulose decomposition (burning of char) between 350 and 600 °C shifted to lower temperatures. The weight loss of the Pt(0)-cellulose specimen took place in nearly one step at 200–250 °C. This is due to the strong catalytic effect of platinum nanoparticles on the oxidation of organic materials [67].

Thus, the decomposition of cellulose hydrogels is in the wide range depending on the cellulose content and the additives. Metal nanoparticles generally increased the thermal stability of the metallogel compared to the initial hydrogel. However, some metals, such as Au or Pt, can be involved in the catalytic processes of cellulose oxidation, especially in air which impacts the thermal stability of the metallogel.

## 3. Chemical Properties

### 3.1. Chemical Composition

Since hydro and metallogels often find their application in the field of biomedicine, their chemical purity is of great importance. They must not contain contaminants and harmful substances. Cellulose hydrogels that were regenerated from the solutions of organic solvents (DMSO, DMAc, etc.) with water rinsing [57] may contain solvent residues in the inner layers because the completeness of the solvent removal is quite difficult to control during the experiment. Elemental analysis and IR spectroscopy make it possible to verify the absence of solvent traces in the ready hydrogels. The elemental analysis of lignocellulosic hydrogels obtained via dissolution in the DMAc/LiCl system detected only C, O, and H in the content of the hydrogels produced from cotton, flax, and deciduous celluloses [64]. However, another study, with the help of energy-dispersive X-ray spectroscopy (EDS), disclosed the trace amounts of N, Na, Cl, Ca, and Mg with a total content of about 1 wt.% in the Ag(0)-cellulose metallogels obtained from the same cellulosic hydrogels according to the same method with the following diffusion-reduction process [72]. Some samples were chemically pure, containing only C, O, H, and Ag. The others were contaminated with inorganic elements in different proportions, which were residuals from the solvent and the reducer trisodium 2-hydroxypropane-1,2,3-tricarboxylate (trisodium citrate). These results indicate that thorough rinsing with deionized water is necessary to obtain chemically pure hydrogels from the solutions of organic solvents. Otherwise, traces of solvent and other excipients may remain in hydro and metal gels.

Another example of the chemical contamination of the ready hydrogels is those produced from waste paper via dissolution in DMAc/LiCl. The hydrogels contained traces of inorganic impurities from additives and ink in the initial paper, but their amount was much less than in the original samples of waste paper [34]. The complete removal of inorganic inclusions from the waste paper solution seems unlikely to happen, so a share of contaminants will be present in the finished hydrogel. As a result, the hydro and metallogels obtained from waste paper cannot be recommended for biomedical purposes. However, there are other possible applications that do not require the complete chemical purity.

### 3.2. Supramolecular Structure

The formation of an organo, hydro, or metallogel leads to the structural changes of cellulose. These transformations can be determined using the X-ray diffraction (XRD) technique. Thus, the native plant-derived lignocellulose samples or waste paper (newsprint paper and cardboard) revealed the crystal structure of cellulose type I allomorph [64,88,108]. However, when analyzing the XRD intensity profiles of the swollen hydrogels, no reflections from the cellulose lattice were observed within the range from 20° to 45° 2θ. Instead, the profiles closely resembled the diffraction pattern typically seen in water. It was due to a very low concentration of cellulose in the hydrogels, namely from 0.5 to 3 wt.%. Freeze-drying of the hydrogels, which is most commonly used for the storage of the hydrogels or to obtain a solid, highly porous structure, resulted in a change in the crystallographic structure of cellulose. The reflections in the XRD profiles corresponded to the structural modification of cellulose II [34,67,87].

Regarding the same structural changes during the hydrogel formation process, Song and co-workers reported bacterial cellulose, which belonged to the crystalline structure of cellulose I. After drying, the XRD peaks of the PVA/BC hydrogels corresponded to the crystalline structure of cellulose II, indicating that BC was dissolved, regenerated, and then produced a lower type II cellulose [75]. PVA/BC-Ag hydrogel obtained via the synthesis of Ag nanoparticles during the hydrogel formation had new XRD peaks at 37.95, 44.13, 64.25, and 77.12° that corresponded to the crystal lattice (111), (200), (220), and (311) planes of silver elements [75].

In the study [68], it was reported that the introduction of silver did not change the supramolecular structure of the hydrogel matrix (cellulose II); however, the diffraction patterns were slightly changed after the intercalation of the gold nanoparticles. This fact does not seem connected to the metal content in the metallogel since both composites with Ag(0) and Au(0) contained a low amount of metals (up to 0.9 wt.% and 1.5 wt.%, correspondingly). With the increase in the content of silver in the metallogels, the crystallinity calculated by the amorphous fitting method slightly increased from 30.6% to 33.8% for the samples containing 0.25% and 0.37% of Ag(0), respectively. There was no noticeable change in the transverse sizes of cellulose crystallites (1.13–1.21 nm) with the increase in Ag(0) content in the metallogels [68].

The additional crystallization occurred during the formation of the composite hydrogel with Zn(II) organic-complex [71]; however, the authors admit the possibility of the change in crystallinity not by the immobilization of 1,10-phencyanine Zn(II) but due to the eventual crystallization of the complex itself in the matrix of the hydrogel. Oppositely, the decrease of the initial cellulosic material’s crystallinity index after introducing Ag particles was reported in [61]. The authors explained this using a bridging bidentate interaction between the COO^−^ groups and Ag^+^ ions. These carboxylate–metal complexes weakened the hydrogen bond system by increasing the distance between the cellulose molecules.

## 4. Biological Properties

### 4.1. Biodegradation

Due to the natural origin of cellulose, its hydrogels are biodegradable substances [78,109]. Cellulose hydro and metallogels can be biodegraded by cellulase, an enzyme synthesized by certain bacteria and fungi species [110]. Several species of microorganisms, namely, *Bacillus subtilis* and *Bacillus licheniformis* from bacteria, and *Pleurotus ostreatus*, *Pleurotus florida*, and *Trichoderma viride* from fungi, are able to synthesize cellulose-specific enzymes (cellulases) in air, water, and soil. Cellulases decrease the molecular weight of cellulose and its mechanical strength and increase solubility, which causes degradation [93,110]. Thus, okara cellulose hydrogels were completely degraded after 28 days due to the microorganisms in the soil [85]. In a soil extraction solution, cellulose hydrogels with 4% of cellulose nanocrystals (CNC) lost 97% of their mass after 10 days of incubation. However, when the content of CNC rose to 5 and 6%, the biodegradability significantly fell to 40 and 35%, respectively [88]. It was explained by the increase in the crosslinking and the decrease in the pore size, which prevented the imbibition of more soil microorganisms in the swollen hydrogel network.

Metallogels retain the ability for biodegradation, though it decreases with the increase of metal content [111]. Of particular importance is the nature of the metal ion, regardless of the use of the metallogel. Even if heavy metal ions complex more intensively with lignocellulosic materials, and the properties of the obtained metallogels are desirable, heavy metals will exert their role as destructive factors on the environment during the following biodegradation; that is why it is preferable to introduce biocompatible ions into the cellulosic substrates [78].

### 4.2. Cytocompatibility

The cellulose hydrogels do not reveal cytotoxic properties due to the natural cytocompatibility of the cellulose matrix and the chemical purity of the hydrogels. For instance, the cytotoxicity assessments showed that the cellulose hydrogels have no cytotoxicity on NIH3T3 and L929 cells [75,84,85]. Being a cytocompatible material, cellulose is also biodurable, which means that bioresorption of cellulose in animal and human tissues does not occur, i.e., cellulose-based materials cannot be digested or metabolized when implanted in vivo [110]. Moreover, the cells, e.g., fibroblasts, are able to grow on the cellulose hydrogel surface, which is a crucial property when the hydrogels are considered for the healing repair application as a scaffold. There was a significant difference in the cell density of the hydrogel films prepared using different dissolving methods. The hydrogel films obtained from DMAc/LiCl solution had the highest cytocompatibility, while the hydrogel films obtained by the NaOH and NaOH/urea methods revealed lower cytocompatibility [74].

Nakason and Kobayashi reported that after the NaOH treatment and NaOCl bleaching, the lignocellulose hydrogel films showed a lower cell density on the surface of the hydrogels (from approximately 20,000 to 38,000 cells/cm^2^) compared to the unbleached ones (about 60,250 cells/cm^2^). Trace lignin is approved for being nontoxic to fibroblast cells [84]. Ag(0) nanoparticles, and generally the other metal nanoparticles, are known to be toxic for eukaryotes in a dose-dependent manner. Nanocomposite dressings with silver nanoparticles impregnated onto a matrix of CNCs were reported to be cytocompatible [112]; the same properties were claimed for the BC hydrogel loaded with silver nanoparticles [62].

Thus, (ligno)cellulose hydrogels and metallogels not only have remarkable biocompatibility b promote fibroblast cell growth. However, the NaOH-based solvents and treatments as well as the high metal content in metallogels, decrease this activity.

### 4.3. Biocidal Properties

Generally, cellolignins fall into the category of substances with potentially biocidal properties [12,113]. Metallogels containing silver, gold, and zinc are known for their antimicrobial and antiviral properties. This effect is associated with the impact of metals, often in the nanophase, against microorganisms. The cellulose matrix does not reduce this effect; on the contrary, it improves the “consumer properties” of the nanoparticles [114]. Cellulose hydrogel acts as a convenient carrier for metal nanoparticles, providing them with suitable mechanical characteristics. Due to the pore system of cellulose hydrogels, a prolonged action of nanoparticles is possible; as a result, the particle release rate can be controlled. Cellulose is biocompatible; with low (but effective) metal content, metallogels are also not toxic to humans [70]. For instance, Ag(0)-cellulose metallogels exhibit broad-spectrum antimicrobial performance against both Gram-positive and Gram-negative bacteria and have a long-acting bactericidal efficacy [58,70,72]. It was shown that silver content in the range of 1–6 wt.% in the metallogels led to the same inhibition of bacterial colonies regardless of the size distribution and content of nanoparticles in the studied interval [72], which is why there is no need to increase the Ag(0) content in a metallogel even if a 1 wt.% is enough.

The BC hydrogels decorated with 4,6-diamino-2-pyrimidinethiol-modified gold nanoparticles show potential in treating bacterially infected wounds. This material demonstrated better efficacy than most of the antibiotics (cefazolin/sulfamethoxazole) against Gram-negative bacteria, while maintaining excellent physicochemical properties including water uptake capability, mechanical strain, and biocompatibility [102]. Thi and co-authors reported strong antibacterial activity of the ZnO-cellulose metallogel toward *Escherichia coli* and *Staphylococcus aureus* at a nanoparticles concentration of 0.025 mg mL^−1^ after 8 h of incubation. The bactericidal activity towards *Staphylococcus aureus* varied extensively with the synthesis parameters [115]. Nanoparticles are sometimes applied together with drugs in order to get a synergistic biocidal effect. For example, the antimicrobial activity of curcumin-loaded hybrid hydrogel with ZnO nanoparticles was proved against *Staphylococcus aureus* and *Trichophyton rubrum* (fungi) [116].

## 5. Conclusions

Cellulose hydrogels possess characteristics such as moisture, softness, smoothness, transparency, and colorlessness. Cellulose-based metallogels preserve the majority of the properties; additionally, they have specific colors depending on the metal component. The color of the composite intensifies as the metal content increases.

Cellulose hydrogels can absorb and retain significant amounts of liquids; the ones obtained via dissolution in DMAc/LiCl exhibit the highest swelling ability, while alkali-based methods result in lower swelling abilities. The swelling behavior of hydrogels is influenced by polymer composition, degree of polymerization, degree of crosslinking, cellulose concentration, cellulose origin, and external stimuli. The introduction of metal nanoparticles can either increase or decrease the swelling ability of the hydrogel. Moreover, the loading of the hydrogels with metal nanoparticles enhances the mechanical strength of the material. However, a high concentration of metal nanoparticles can deteriorate mechanical properties due to particle agglomeration.

Formation of hydrogels leads to a change in the supra-molecular structure of cellulose, converting it from the crystalline structure of cellulose I to a lower type II cellulose after dissolution. The introduction of metal into the hydrogel generally does not alter the supramolecular structure of the cellulose matrix, but it does increase the crystallinity index with higher metal content. However, some studies have shown a decrease in the initial crystallinity index of cellulosic material after decoration with metal particles.

The decomposition rate of cellulose hydrogels varies depending on the cellulose content and additives. Metal nanoparticles generally enhance the thermal stability of metallogels compared to initial hydrogels. Nevertheless, certain metals, such as gold or platinum, can participate in catalytic processes, e.g., cellulose oxidation, especially in the presence of air, which can affect the thermal stability of the metallogel.

Cellulose is considered a resistant biomaterial, which through biodegradation, is susceptible to the environment. Metallogels containing silver, gold, and zinc oxide are known for their antimicrobial and antiviral properties. The porous structure of the cellulose facilitates the ionic exchange of metal ions and the formation by complexation of some metallogels, with the potential biocidal function with a prolonged effect.

The incorporation of metal ions into cellulose-based gels introduces unique properties and functionalities. Metal ions or metal nanoparticles can impart new desirable characteristics, such as optical responsiveness, thermal properties, or biological stability, to the gel matrix, expanding the range domain of potential applications. Further research in this field holds great promise for exploring their full potential and expanding their range of applications.

## Figures and Tables

**Table 1:** Properties of the cellulose hydrogels.

Initial Material	Solvent	(Ligno)Cellulose Concentration in the Solution, %	Swelling Ability, g/g	Physico-Chemical Properties	Reference
Bamboo fibers	DMAc/LiCl (hydrogel films)	1	0.31	Tensile strength 66 N/mm^2^, Elongation 33.5%	[64]
	NaOH/ water (hydrogel films)		0.14	Tensile strength 27 N/mm^2^, Elongation 13%	
	NaOH/urea (hydrogel films)		0.11	Tensile strength 21 N/mm^2^, Elongation 8%	
Okara cellulose	LiOH/ urea/water (8:15:77), ECH	6	0.88–0.94	Tensile strength 0.024–0.245 N/mm^2^, Elongation 60.5–107%, Young’s modulus 0.0087–0.37 mpa	[73]
Flax lignocellulose	DMAc/LiCl	1–3	25	Porosity 98.9%	[52]
Hardwood lignocellulose			28	97.4%	
Cotton MCC			20	86.8%	
Sugarcane bagasse lignocelluloses	DMAc/LiCl (hydrogel film)	1	11.53–15.25	Tensile strength 0.43–0.80 N/mm^2^, Elongation 26.5–45.2%	[72]
Cellulose	NMMO	3	3.94	T_decompos_. 335 °C	[80]
		8	6.19	352 °C	
Thanaka heartwood cellulose	DMSO/LiCl (hydrogel film)	1	1.66	Elongation 9%	[74]
Sugarcane bagasse cellulose			1.89	12.45%	
Rice straw cellulose			1.69	35.71%	
Sago pith waste cellulose	NaOH/urea aqueous (7:12), ECH	3	2.4	T_decompos_. 300–400 °C	[63]
		4	2.0		
		5	19.7		
		6	8.1		
Waste paper/acrylamide	NaOH/urea aqueous (7:12), ECH	2	66.75	Porosity 98.1% T_decompos_. 407,72 °C	[75]
Waste office paper	NaOH/urea/ water (7:12:81), ECH	4	18.0		[76]
		5	27.7		
		6	13.4		
Soybean stem lignocellulose with lignin content: 23.24%	DMSO/LiCl, EDA	5	at 25 °C 22.87	Average pore size 20.281 nm	[82]
21.33%			11.29	16.301 nm	
18.91%			7.71	16.709 nm	
14.24%			13.96	17.813 nm	
PVA/BC	NaOH /urea/water	4	13	Tensile strength 0.051 N/mm^2^, elongation 160%	[65]
PVA/BC/AgNO_3_			16	Tensile strength 0.2 N/mm^2^, elongation 180%	

MCC—microcrystalline cellulose, ECH—epichlorohydrin, a crosslinking agent; NMMO—N-methylmorpholine-N-oxide, PVA—polyvinyl alcohol, BC—bacterial cellulose, EDA—ethylenediamine.

## Data Availability

Not applicable.
